# Supplementary material for: Association between phase angle, body mass index and insulin resistance in patients with type 2 diabetes mellitus: a cross-sectional study
Source: PeerJ. 2025 Jan 16;13:e18815. doi: 10.7717/peerj.18815 (PMC11742247; doi:10.7717/peerj.18815)
Supplement: Supplemental Information 2 [file peerj-13-18815-s002.docx]

Dear Editor, our ethics document already contains a section on free informed consent, which I have highlighted in yellow and annotated in English in the attached document.

Regarding the id in our raw data is not really an ID number, it's a number we sort by. We've set the id into No. We've re-uploaded the raw data file.

The categorical variables in the raw data were explained as follows:

SEX column：1 for male， 2 for female

FZ column：1 for low phase angle and low body mass index (LPLB)

2 for low phase angle and high body mass index (LPHB)

3 for high phase angle and low body mass index (HPLB)

4 for high phase angle and high body mass index (HPHB)

Thank you for your question and I hope my manuscript will meet the requirements of your journal!
